# Honeysuckle Extracts (*Lonicera japonica* Thunb.): Understanding Insights into the Antioxidant Effect on Preserving Qualities of Rabbit Meat During Refrigerated Storage

**DOI:** 10.3390/foods14244194

**Published:** 2025-12-06

**Authors:** Xiaohua Huang, Wenjiao Chen, Siyi Yang, Bo Wang, Xinyu Hu, Zhilan Dong, Jiamin Zhang

**Affiliations:** 1 Meat Processing Key Laboratory of Sichuan Province, College of Food and Biological Engineering, Chengdu University, Chengdu 610106, China; 2 Sichuan Provincial Engineering Research Center of Meat Quality Improvement and Safety Control Technology, Chengdu 610106, China; 3 Bahotu Food Development Co., Ltd., Zigong 643000, China

**Keywords:** honeysuckle extract, shelf-life, rabbit meat, oxidation

## Abstract

This study aimed to evaluate the potential of honeysuckle extract (HE) as a natural preservative for rabbit meat. We investigated its antioxidant and antibacterial properties and its effect on the quality of pre-treated rabbit meat during refrigerated storage (4 °C) for up to 15 days. Firstly, honeysuckle was extracted using ethanol at different concentrations (50–75%). Extraction with 65% ethanol yielded optimal antioxidant activity and a high content of bioactive compounds. Subsequently, both raw and cooked rabbit meat, pre-treated with a standard marinade, were treated with 4% HE and stored for 15 days. The results showed that HE treatment significantly stabilized chewiness compared to the control, although no significant differences were observed in hardness, springiness, or cohesiveness. Moreover, HE treatment significantly reduced TBARS, TVB-N, and TVC levels (*p* < 0.05) compared to the control group. Taken together, HE could inhibit microbial proliferation, and lipid oxidation in pre-treated rabbit meat during refrigerated storage, indicating HE as a potential natural preservative for improving quality loss and prolonging the shelf-life of refrigerated rabbit meat.

## 1. Introduction

Meat has been an essential source of high-quality protein and numerous nutrients in the human diet [1]. Consumption of high animal protein and low-fat content is associated with enhanced human nutrition, growth, development, and health [2]. Rabbit meat is an excellent source of high protein (21–23%), low cholesterol, high levels of essential fatty acids, and other bioactive compounds [3,4], and is a preferred meat for high-level meat consumers, older people, and bodybuilders [5,6]. Yet, the high polyunsaturated fatty acid (PUFA) and protein content are vulnerable to oxidative attack during storage via autoxidation and enzymatic pathways [7,8]. This could lead to many problems, such as rancidity, discoloration, nutrient loss, and diminished economic value of rabbit meat products.

To solve these problems, some synthetic or artificial additives, such as butylated hydroxyanisole (BHA) and butylated hydroxytoluene (BHT), are commonly employed in meat preservation [9]. These synthetic additives effectively prolong the shelf-life and preserve the quality of rabbit meat. However, concerns regarding the safety of some synthetic or artificial additives have led to restrictions or bans in some countries, due to their association with potential health risks [10]. Meanwhile, the growing consumer demand for clean-label and naturally preserved products has intensified the search for safer alternatives that effectively preserve rabbit meat and its product quality [11].

Plant extracts, recognized as natural sources of potential food additives, offer advantages due to their simple preparation and strong antioxidant capacity, allowing for them to be safely used in food preservation [12,13]. Extensive research has focused on plant extracts in various meats (beef, chicken, lamb, etc.) and animal products [14,15]. Some plant aqueous extracts, such as *Curcuma longa* L., *Myristica fragrans* Houtt., *Zingiber officinale* Roscoe, *Cymbopogon citratus* (DC.) Stapf, and *Thymus vulgaris* L., could reduce the thiobarbituric acid reactive substances (TBARS) value and extend the shelf-life of cold-stored rabbit meat. Among them, *Zingiber officinale* exhibited the highest DPPH scavenging activity and *Myristica fragrans* the strongest ABTS scavenging activity, while a synergistic mixture of all extracts proved most effective by suppressing lipid oxidation and increasing shelf life by 50% [16]. A recent study showed that purslane extract, which exhibited potent radical scavenging activity with IC_50_ values of 5.112 ± 0.384 μg/mL and 12.607 ± 2.130 μg/mL for DPPH and ABTS assays, respectively, effectively inhibited the rates of lipid and protein oxidation in rabbit meat [17]. This result was explained by the potent bioactive compounds found in the extracts, including polyphenols, flavonoids, and organic acids, all of which have been exhibited to have antioxidant, anti-inflammatory, and antibacterial properties [18].

Honeysuckle (*Lonicera japonica* Thunb.), an excellent plant resource that has the concomitant function of both medicine and food, exhibits anti-inflammatory, antioxidant, antiviral, and anti-cancer properties [19]. Honeysuckle has been identified as comprising several kinds of chemical components, including polysaccharides, flavonoids, volatile oils, organic acids, and iridoid glycosides [20]. These compounds exhibit various bioactive properties, such as free radical scavenging activity, metal ion chelation capacity, and enzyme inhibition properties, which can prevent oxidation and discoloration, as well as inhibit microbe growth [21,22]. A recent study showed that honeysuckle leaf extract in the bioactive edible films had higher DPPH and ABTS scavenging activity and inhibited microbe growth, such as *Escherichia coli*, *Listeria monocytogenes*, and *Staphylococcus aureus* [23]. Thus, HE is a potential candidate for a natural antioxidant. However, HE’s effectiveness as a natural preservative in rabbit meat is still unclear, even though it is a strong antioxidant and limits the growth of microorganisms.

Therefore, this study aims to comprehensively study the antioxidant and antibacterial properties of HE and its impact on the preservation of pre-treated rabbit meat during refrigerated storage. The study encompasses two main components: (1) Investigation of the effect of ethanol concentration on the content of bioactive compounds and antioxidant activities of HE. (2) Evaluation of the efficacy of HE in preservation pre-treated rabbit meat. The study is essential for developing HE as a potential natural preservative for prolonging the shelf-life of refrigerated rabbit meat.

## 2. Materials and Methods

### 2.1. Preparation of HE

The dried flowers of honeysuckle samples (Nanjiang Liangyuan Food, Bazhong, China) were washed and air-dried in an electric heating constant-temperature forced-air-drying oven. The dried samples were subsequently ground into a fine powder and passed through a 60 mesh sieve in preparation for ethanol extraction. The ethanol used contained different proportions and was hypothesized to influence the efficiency of the polyphenol extraction, following the methods described by Opris et al. with slight modifications [24]. In brief, 5 g of powdered honeysuckle were accurately weighed into a round-bottom flask, with six different concentrations of ethanol solvents (50%, 55%, 60%, 65%, 70%, and 75% *v*/*v*) added at a solid-to-liquid ratio (S/L) of 1:25 (*w*/*v*). Extraction was performed using a reflux extraction technique at 65 °C with 60 rpm for 1 h. The supernatant was gathered by filtering, and the residual powder was repeated twice more.

### 2.2. Total Phenolic (TPC), Flavonoid (TFC), and Chlorogenic Acid (CGA) Contents Measurement

The TPC and TFC were measured by the Folin–Ciocalteu reagent and the aluminum nitrate method, using gallic acid and rutin as the standard, respectively. Briefly, the sample or standard solution was prepared as a test solution with an appropriate concentration using an ethanol–water solution. For TPC, the test solution was added with an equal volume of Folin–Ciocalteau and 20% Na_2_CO_3_ (*w*/*v*). The mixture was vortexed and subsequently heated in 50 °C water for 40 min. The absorbance was measured at 760 nm using a UV-1100 spectrophotometer (Mapada Instruments, Shanghai, China). The TPC was calculated from the known standards multiplied by the dilution factor, and the results are expressed as milligrams of gallic acid equivalent (mg GAE/mL extract). For TFC, the test solution was added to 10 μL of 5% NaNO_2_ (*w*/*v*) at room temperature for 6 min. Then, 10 μL of 10% Al(NO_3_)_3_ was added, mixed, and left for 6 min. The mixture was added to 20 mL of 4% NaOH, mixed, and subsequently left for 15 min. The absorbance was measured at 510 nm by spectrophotometer (UV-1100). The TFC was calculated from the known standards, multiplied by the dilution factor, and the results are expressed as milligrams of rutin equivalent (mg RE/mL extract).

The CGA content was measured via ultraviolet–visible spectrophotometry and using chlorogenic acid as the standard. The absorbance was measured at 329 nm by spectrophotometer (UV-1100). The chlorogenic acid content was calculated from the known standards, multiplied by the dilution factor.

### 2.3. DPPH and ABTS Assay

DPPH and ABTS radical scavenging rate activity were performed with their commercial assay kits (Solarbio Science & Technology, Beijing, China), respectively, according to the manufacturer’s instruction. Vitamin C (Vc) was used as a positive control in this experiment. Briefly, the samples were diluted 5-fold with the extraction solution. Samples of 10 µL and Vc solution were added into a 96-well transparent plate and were reacted with 190 µL DPPH or ABTS working solution. The plate was incubated at 37 °C in the dark for 30 min and 6 min for the DPPH and ABTS assays, respectively. The absorbance was measured at 515 nm (DPPH) and 405 nm (ABTS) on a plate reader (FlexA-200, Molecular Devices, Hangzhou, China).

### 2.4. Preparation of Rabbit Meat

Rabbit meats were obtained from Ha Wu Ye Food Co., Ltd. (Leshan, Sichuan, China), which were cut into pieces (3 cm × 3 cm × 3 cm) after washing. The rabbit meat samples were marinated with salt, cooking wine, starch, monosodium glutamate, and phosphate compound water-retaining agent, then divided into control (treated with deionized water: control for raw (RC), control for cooked (CC)) and treated groups (treated with 4% HE: HE for raw (RHE), HE for cooked (CHE)). All samples were kneaded for 10 min and placed at 4 °C for 1 h. The cooked groups (CC and CHE) were subsequently boiled at 100 °C for 10 min, cooled down, and we removed the surface water with filter tissue. All samples were vacuum-packed and stored at 4 °C for 15 days, and samples were taken every 3 days for analyses.

### 2.5. Determination of Moisture Content, Water Activity (Aw)

For moisture measuring, 2 g of a rabbit meat sample was placed into the sample dish in ambient conditions at room temperature and the humidity of 60%. The moisture content value was analyzed by a moisture content tester WL-70F (Guanya, Shenzhen, China). The Aw values were measured according to the Chinese National Food Safety Standard GB 5009.238-2016 [25]. The measurement was performed using a water activity meter, based on the diffusion method. Briefly, 1 g of sample was left on a transparent plate in ambient conditions, and the Aw value was measured at 5 min intervals by water activity meter HD-5 (Huake, Wuxi, China) at room temperature and a humidity of 60%. Each sample was analyzed in triplicate following instrument stabilization.

### 2.6. Color and Texture Profile Analysis (TPA)

The color parameters (L*, a*, b*) of rabbit meat were measured using a calibrated chromameter (CR-400, Konica Minolta, Tokyo, Japan). The TPA was performed using a TA-XT Plus texture analyzer (Stable Micro Systems Ltd., London, UK) with a 36 mm cylindrical probe (P/36). Samples (1 cm × 1 cm × 1 cm) were analyzed under the following conditions: pre-test speed = 1 mm/s, test speed = 1 mm/s, and trigger force = 5 g.

### 2.7. Thiobarbituric Acid Reactive Substance (TBARS) Assay

The TBARS content was detected according to the spectrophotometric method in the Chinese National Food Safety Standard GB 5009.181-2016 [26], with slight modifications. Briefly, 5 g of rabbit meat was homogenized in 50 mL of 7.5% trichloroacetic acid (*w*/*v*) mixture with a shaker at 50 °C for 30 min. A total of 5 mL of the filtrate or standard was transferred to a new colorimetric tube and mixed with 5 mL of 0.02 mol/L thiobarbituric acid solution. The mixture was subsequently heated at 90 °C for 30 min. The absorbance was measured at 532 nm by spectrophotometer (UV-1100). The TBARS value was calculated from the known standards, multiplied by the dilution factor.

### 2.8. Total Volatile Basic-Nitrogen (TVB-N) Assay

The TVB-N content was measured following the Chinese National Food Safety Standard GB 5009.228-2016 (semi-micro nitrogen method) with slight modifications [27]. Briefly, 20 g of rabbit meat was vortexed in 100 mL distilled H_2_O, impregnated, and filtered. A total of 10 mL of filtrate was added to 5 mL magnesium oxide and was distilled for 5 min using a Kjeldahl Apparatus (KDN-102C, Shanghai xianjian instruments Co., Ltd., Shanghai, China). The distillate was titrated with 0.01 mol/L HCl. The TVB-N content was calculated as follows: TVB-N (mg/100 g) = [(V_1_−V_2_) × c × 14]/[m × (V−V_0_)] × 100, where V_1_ and V_2_ represent HCl volumes (mL) for sample and blank, respectively; V and V_0_ represent the aliquot filtrate volume and total filtrate volume, respectively; and c and m represent the concentration of HCl standard titration solution and the mass of sample, respectively. Distilled H_2_O was used as a blank control in this experiment.

### 2.9. Microbiological Analysis

Microbiological analyses were evaluated according to the Chinese National Food Safety Standard GB 4789.2-2022 [28]. Briefly, 25 g of rabbit meat samples were mixed with 225 mL phosphate-buffered saline under aseptic conditions. The total viable count (TVC) was measured by evenly mixing appropriately diluted sample solutions with plate count agar medium and incubated at 36 ± 1 °C for 48 ± 2 h.

### 2.10. Statistical Analysis

Data were analyzed with SPSS 26 software (Cary, NC, USA). The independent *t*-test was employed to compare differences between two groups. A one-way analysis of variance (ANOVA) procedure, followed by Tukey’s multiple range, was applied to analyze the difference between the groups, when two-way ANOVA was used to analyze the effect of HE on the texture profile of rabbit meat. Pearson correlation coefficients were calculated to evaluate the relationships between the variables. The correlation heatmap, combined with Mantel test results, was visualized using the ggcor package in R-4.5.1 software. The results were presented as mean ± SE, and statistical significance was determined at *p*-value less than 0.05. The study was replicated three times, and three measurements were conducted per replicate.

## 3. Results

### 3.1. Total Phenolic, Flavonoids, and Chlorogenic Acid Content of the HE

**Figure 1:**
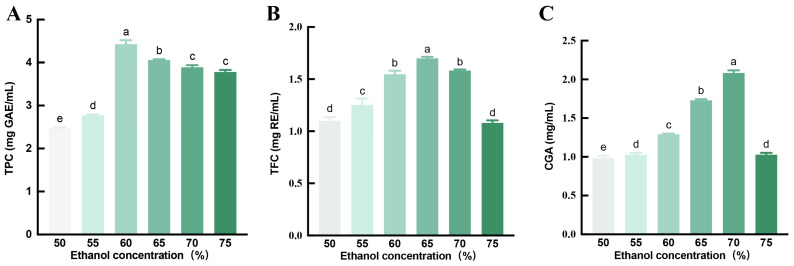
Effect of extraction with different concentrations of ethanol solvents on bioactive compounds of honeysuckle. (**A**) TPC contents in the extracts. (**B**) TFC contents in the extracts. (**C**) CGA contents in the extracts. (*N* = 3 for each group). Data are expressed as mean ± SE. Different lowercase letters (a–e) indicate the significant differences (*p* < 0.05) among groups.

Previous studies found that natural antioxidants derived from plants, such as phenolic compounds, exhibit varying solubility profiles, typically favoring low-polarity organic solvents like methanol, ethanol, and acetone, often with specific water content adjustments [29]. The results varied with the concentration of ethanol solvents used [30]. To investigate the effect of ethanol concentration on the content of bioactive compounds, the concentration of ethanol solvents with 50% to 75% *v*/*v* were used to extract the TPC, TFC, and CGA of honeysuckle. The results showed that the TPC, TFC, and CGA content exhibited a parabolic trend across the tested ethanol concentrations (Figure 1). Maximum yields were observed at 60% ethanol for TPC (4.05 mg GAE/mL), 65% ethanol for TFC (1.70 mg RE/mL), and 70% ethanol for CGA (2.08 mg/mL). Notably, extraction with 75% ethanol resulted in significant reductions of 14.9%, 36.6%, and 50.7% in TPC, TFC, and CGA content, respectively, compared to their optimal values. The results suggest that ethanol concentration is a key parameter governing the efficiency and selectivity of bioactive compound extraction in the extraction process from honeysuckle.

### 3.2. Antioxidant Activity of the HE

**Figure 2:**
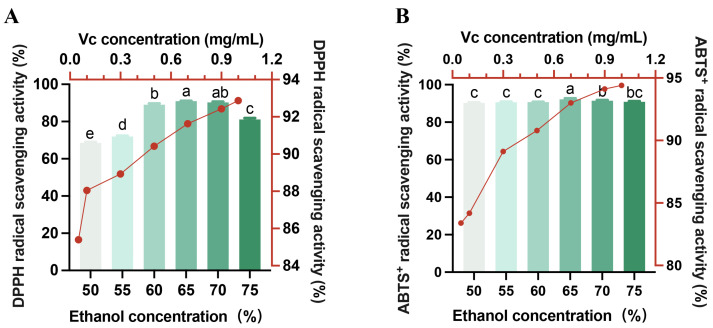
Antioxidant activity of honeysuckle in different concentrations of ethanol solvents. (**A**) DPPH radical scavenging activity, (**B**) ABTS radical scavenging activity. (*N* = 3 for each group). Data are expressed as mean ± SE. Vc, vitamin C. Different lowercase letters (a–e) indicate the significant differences (*p* < 0.05) among groups.

The antioxidant activities of HE were then evaluated under different ethanol concentrations (50–75% *v*/*v*) and extraction conditions. The results revealed that the DPPH and ABTS radical scavenging capacities exhibited a concentration-dependent response (Figure 2). The ability of the extracts to scavenge the DPPH and ABTS radicals first increased, followed by a decline in conjunction with a higher ethanol volume fraction, reaching a maximum of 65% (90.92% and 92.19%, respectively).

Because 70% or 75% ethanol extraction had a similar or decreased effect on the DPPH and ABTS radical scavenging capacities of the honeysuckle with 65% ethanol extraction, 65% ethanol extract of honeysuckle was selected as a follow-up study on efficacy for raw or pre-treated rabbit meat preservation.

### 3.3. Effect of HE on the Aw and Moisture Content of Pre-Treated Rabbit Meat During Refrigerated Storage

**Figure 3:**
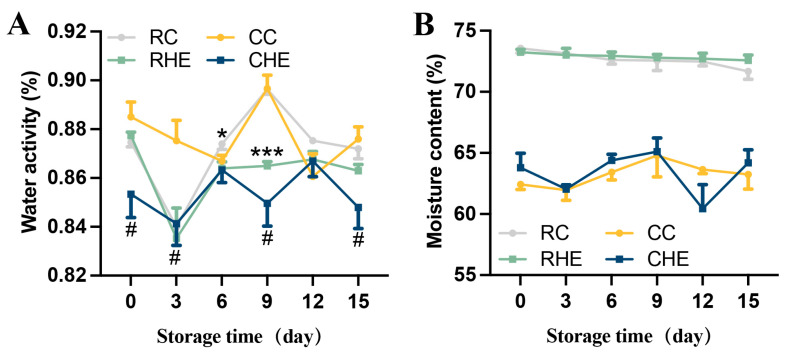
Changes in the Aw and moisture content of raw and cooked pre-treated rabbit with HE treatment during refrigerated storage. (**A**) Water activity, (**B**) moisture content. (*N* = 3 for each group). Data are expressed as mean ± SE. # or * values indicate significant differences within the same storage time. # *p* < 0.05 CC vs. CHE. * *p* < 0.05, *** *p* < 0.001 RC vs. RHE. RC, control for raw; CC, control for cooked; RHE, HE for raw; and CHE, HE for cooked.

To explore the role of HE in the raw and cooked pre-treated rabbit meat preservation, raw and cooked marinated rabbit meat were treated with 4% HE during processing and stored at 4 °C for 15 days. Results showed that during the storage period, in cooked pre-treated rabbit meat, CHE group exhibited lower water activity than CC group on days 0, 3, 9, and 15 (Figure 3A), even though the moisture contents were not significantly different between the two groups (Figure 3B). Similarly, in raw pre-treated rabbit meat, HE-treated meat also had decreased water activity on days 6 and 9, though the moisture content did not change compared with the control (Figure 3A,B). These results indicated that HE treatment could suppress water activity in both raw and cooked pre-treated rabbit meat, independent of moisture alteration.

### 3.4. Effect of HE on Color of Pre-Treated Rabbit Meat During Refrigerated Storage

**Figure 4:**
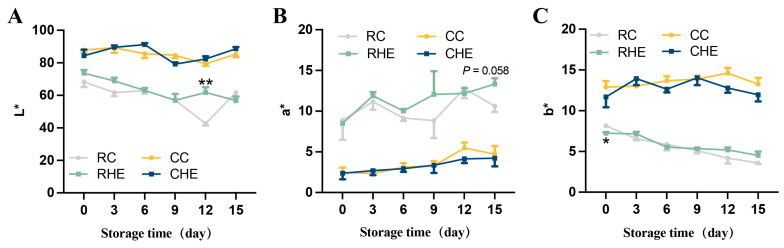
Changes in the color of raw and cooked pre-treated rabbit with HE treatment during refrigerated storage. (**A**) L* value, (**B**) a* value, (**C**) b* value. (*N* = 3 for each group). Data are expressed as mean ± SE. * Values indicate significant differences within the same storage time. * *p* < 0.05, ** *p* < 0.01, RC vs. RHE. RC, control for raw; CC, control for cooked; RHE, HE for raw; and CHE, HE for cooked.

Color is an important sensory feature that affects consumer opinions and choosing meat products [31]. Thus, the color was then evaluated in the raw and cooked pre-treated rabbit meat. Changes in the surface color of raw and cooked pre-treated rabbit meat treated without and with 4% HE during 15 days of refrigerated storage are shown in Figure 4. For raw rabbit meat, the L* values were only significantly increased by HE treatment on day 12 of the storage period as compared with the control group, which might be due to antioxidant protection of myoglobin from oxidation in rabbit meat. For a* values, no change was observed between HE-treated and control groups from days 0 to 12, while the a* values tended to increase in the HE-treated group on day 15 of the storage period. Furthermore, on day 0, the HE-treated group had lower b* values than the control group. However, it was noted that the b* values from days 3 to 15 were similar between the two groups. For cooked pre-treated rabbit meat, no significant difference was observed in the HE-treated group during the storage period on L*, a*, and b* values, which were in the range from 77.15 to 95.24, 1.2 to 6.47, and 10.17 to 15.83, respectively.

### 3.5. Effect of HE on Texture Profile of Pre-Treated Rabbit During Refrigerated Storage

The textural profiles and the taste of rabbit meat can be affected by the loss of water, microbial action, and protein degradation during the refrigeration process. The textural profiles, including hardness, elasticity, chewiness, and cohesiveness were then analyzed in raw and cooked pre-treated rabbit meat without and with 4% HE treatment during 15 days of refrigerated storage. As shown in Table 1, the HE-treated and control groups showed a similar tendency of variation in hardness, elasticity, and cohesiveness with the extension of refrigerated storage under raw or cooked pre-treated conditions. As for chewiness, the RC and RHE groups exhibited a significant downward trend during refrigerated storage. Furthermore, HE treatment significantly increased the chewiness at days 3, 6, and 9 in raw pre-treated rabbit meat, as compared to those in the control groups. A similar trend was observed at days 3 and 6 for cooked pre-treated rabbit meat. The results suggested that HE could stabilize the chewiness of pre-treated rabbit meat under raw or cooked conditions.

### 3.6. Effect of HE on Lipid Oxidation of Pre-Treated Rabbit During Refrigerated Storage

**Figure 5:**
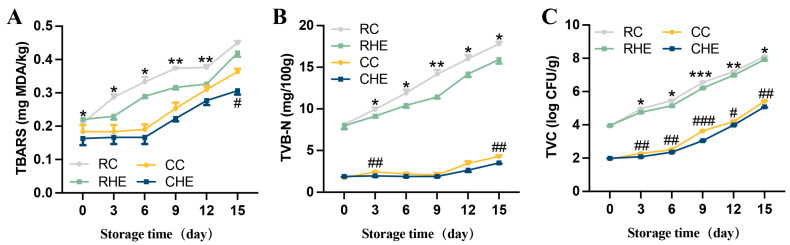
Changes in the quality preservation and oxidation profiles of raw and pre-treated rabbit with HE-treated during refrigerated storage. (**A**) TBARS content, (**B**) TVB-N content, (**C**) TVC content. (*N* = 3 for each group). Data are expressed as mean ± SE. # or * values indicate significant differences within the same storage time, CC vs. CHE and RC vs. RHE, respectively. #, * *p* < 0.05, **, ## *p* < 0.01, ***, ### *p* < 0.001. RC, control for raw; CC, control for cooked; RHE, HE for raw; and CHE, HE for cooked.

The TBARS value is commonly used as an indicator for evaluating the degree of lipid oxidation in meat products. Higher TBARS levels indicated a greater extent of lipid oxidation. The change in TBARS value of raw and cooked pre-treated rabbit meat without and with 4% HE treatment during 15 days of refrigerated storage are shown in Figure 5A. The TBARS values of the RC, RHE, CC, and CHE groups increased, accompanied by growing storage duration, revealing that lipids in rabbit meat are continually oxidized during the refrigeration process. For raw pre-treated rabbit meat, the TBARS values were significantly decreased by HE treatment during days 0 to 12 of storage compared with those in the control groups. For cooked pre-treated rabbit meat, the levels of TBARS were similar between the HE-treated and control groups during days 0 to 12 of storage. However, when the storage time was increased to day 15, CHE groups had lower TBARS values compared with the control group. These results indicated that HE might prevent lipid oxidation in both raw and cooked pre-treated rabbit meat during refrigerated storage.

### 3.7. Effect of HE on Antimicrobial Activity of Pre-Treated Rabbit During Refrigerated Storage

TVB-N is an important indication to evaluate the freshness of meat products. As shown in Figure 5B, for raw pre-treated rabbit meat, HE-treated groups had lower TVB-N levels during days 3 to 15 of storage than those in the control groups. For cooked pre-treated rabbit meat, the level of TVB-N in the HE-treated group decreased by 19.4% and 18.2% at days 3 and 15, respectively, compared with those in the control group.

Next, TVC levels were investigated in raw and cooked pre-treated rabbit meat. On day 0, no significant difference was observed in HE-treated meat during the storage period in the TVC levels for raw and cooked pre-treated rabbit meat, which were in the range from 3.87 to 4.04 log CFU/g, and 1.91 to 2.01 log CFU/g, respectively (Figure 5C). However, for raw pre-treated rabbit meat, HE-treated meat significantly reduced TVC levels with prolonged storage time compared to the control group. Generally, TVC values below 4.00 log CFU/g could indicate that the microbiological quality of cooked meat products is allowable [32]. For cooked pre-treated rabbit meat, the TVC of CC groups reached the threshold limit at day 12, while the CHE groups became unacceptable until day 15, with the TVC levels of 4.20 and 5.08 log CFU/g, respectively. These data suggested that HE effectively inhibited microbial growth in pre-treated rabbit meat and extended the refrigerated shelf-life.

### 3.8. Correlation Analysis

**Figure 6:**
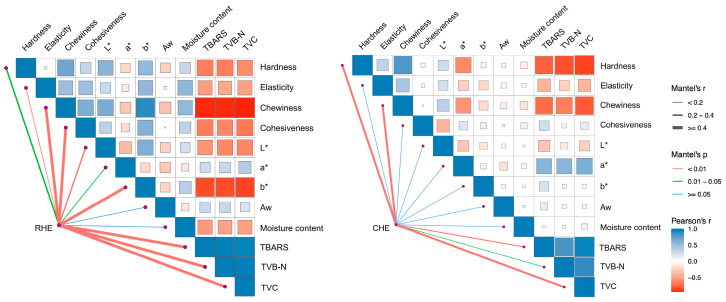
Correlation analysis of quality parameters in raw and pre-treated rabbit with HE treatment during refrigerated storage. The pairwise comparisons within the heatmap display the Pearson correlation coefficients. The color gradient represents the correlation strength: blue indicates a positive correlation, while red indicates a negative correlation. The size of the square corresponds to the magnitude of the correlation coefficient.

To further elucidate the interrelationships between physicochemical properties, microbial growth, and textural attributes during storage, Pearson correlation analyses were conducted. As shown in Figure 6, a consistent pattern was observed in both raw and cooked pre-treated rabbit meat. There were strong positive correlations (indicated by blue squares) among TBARS, TVB-N, and TVC. This significant positive relationship confirms that lipid oxidation, protein degradation, and microbial proliferation occurred synchronously during refrigerated storage. Conversely, distinct negative correlations (indicated by red/orange squares) were observed between these spoilage indicators (TBARS, TVB-N, and TVC) and texture profile parameters, particularly hardness and chewiness.

## 4. Discussion

The current study reported that HE supplementation could affect oxidation in raw and pre-treated rabbit meat during refrigerated storage. Firstly, honeysuckle extracted with 65% ethanol showed strong antioxidant activity (DPPH and ABTS) and the optimal content of active compounds (TPC, TFC, and CGA). In addition, 4% HE-treated could delay the textural deterioration in pre-treated rabbit meat during refrigerated storage. Furthermore, 4% HE treatment could inhibit the microbial growth and lipid oxidation in pre-treated rabbit meat during refrigerated storage.

Oxidative stress plays a role in the development of a variety of diseases [33,34]. Honeysuckle has been used in traditional Chinese medicine herbs and contains numerous bioactive compounds with reported antioxidant and anti-inflammatory activities [19,35]. The antioxidant activity of crude plant extracts and purified compounds has been investigated using the DPPH and ABTS free radical scavenging activity assessments [36,37]. The current study reported that honeysuckle extracted with 65% ethanol showed the optimal content of TFC and the suboptimal levels of TPC and CGA, which directly coincided with the maximum DPPH and ABTS free radical scavenging activities, indicating a strong positive correlation between the content of these active compounds and the antioxidant potential of the extract. Moreover, previous studies revealed a positive relationship between active compounds and antioxidant activity in several plants and foods [30]. Rice-Evans et al. reported that the total antioxidant activity correlates with elevated levels of phenolic compounds in foods and beverages [38]. Seo et al. reported that the antioxidant activity was dependent on flavonoid content, with the leaf exhibiting the highest activity, which correlated with the highest concentration of flavonoids in honeysuckle [39]. Furthermore, the current study showed that the decline in antioxidant capacity at higher ethanol concentrations (70 or 75%) parallels the reduced extraction of TPC and TFC, confirming that the abundance of these hydrogen-donating antioxidants determines the scavenging efficacy of the honeysuckle extract. Thus, both extremely low and high ethanol concentrations were not ideal for the extraction of the target bioactive compounds from honeysuckle. When the organic solvent content is low, the enhanced polarity of the solvent system may result in compromised selectivity toward dissolving hydrophilic impurities, such as soluble proteins, water-soluble vitamins, and sugars. In contrast, too high organic solvent concentrations significantly increase system viscosity, inhibiting the dissolution rate of target compounds, leading to lower extraction efficiency [40,41]. In addition, the ethanol concentration-dependent trends observed for DPPH and ABTS radical scavenging activities closely linked those of the TPC, TFC, and CGA contents. However, a more comprehensive comparison between HE and synthetic or artificial additives, such as BHT or HBA, including antioxidant properties, needs further study to be explored.

Lipid oxidation during refrigerated storage compromises nutritional quality, accelerates meat deterioration, and impairs flavor via MDA formation, which is a key secondary peroxidation product quantified as TBARS [42]. During storage, constant oxidation in meat products generates secondary oxidation products (aldehydes, ketones, acids) that drive TBARS accumulation [43]. Wu et al. found that TBARS value had a positive correlation with the storage duration in rabbit meat, indicating continuous oxidation during storage [44]. Here, the current study showed that the TBARS values significantly increased during the 15 days of refrigerated storage. Similar findings were also reported [45]. In addition, we showed that the HE treatment decreased TBARS levels in pre-treated rabbit meat during refrigerated storage. Generally, enzymatic oxidation and non-enzymatic oxidation are the two main types of lipid oxidation. The key enzymes, including lipoxygenase, superoxide dismutase (SOD), and glutathione peroxidase (GPx), are inactivated during the cooking process. Thus, non-enzymatic oxidation, such as autoxidation and photo-oxidation, have become the primary pathways of lipid oxidation in cooked meat. Furthermore, PUFA in meat undergo oxidation triggered by heat, metal ions, and reactive oxygen species, initiating a radical chain reaction that forms hydroperoxides and secondary oxidation products [43]. Furthermore, the lower TBARS values in HE-treated rabbit meat correspond to the synergistic antioxidant function of active compounds in honeysuckle (Figure 1), which reduce oxidation processes by quenching free radicals in meat products through proton or electron donation [46]. Therefore, HE exerted a potential decelerated oxidative deterioration effect on pre-treated rabbit meat during refrigerated storage by regulating the antioxidant activity of active compounds. In addition, the antioxidant efficacy of HE may also be attributed to other protective mechanisms, such as transition metal chelation and the modulation of intracellular antioxidant signaling pathways. Transition metal ions, particularly Fe^2+^ and Cu^2+^, are potent catalysts for lipid peroxidation in meat systems. Bioactive flavonoids in honeysuckle possess hydroxyl and carbonyl groups that are capable of chelating these pro-oxidant ions, thereby forming inactive complexes and blocking the initiation of radical chain reactions [47,48]. Furthermore, other studies indicate that *Lonicera japonica* polyphenols can activate the nuclear factor erythroid 2-related factor 2 (Nrf2) pathway, upregulating endogenous antioxidants for sustained cellular defense [49]. It is interesting to figure out the other mechanism effects of HE on antioxidants in rabbit meat, which will be explored in future studies.

TVB-N is considered to be a prominent indicator of meat deterioration and mostly consists of ammonia and amines produced when endogenous enzymes and spoilage bacteria break down animal proteins [50]. Herein, we showed that HE treatment exhibited lower TVB-N levels in pre-treated rabbit meat during refrigerated storage. According to the Chinese national food safety standard GB2707-2016 [51], the acceptable limit for TVB-N values lower than 15 mg/100 g is indicated as a threshold of high freshness. The current study showed that all groups had similar TVB-N levels, which range from 7.55 to 8.83 mg/100 g in raw pre-treated rabbit meat at day 0, indicating the good quality of raw pre-treated rabbit meat. In addition, the TVB-N values of the RC exceeded the acceptable limit at day 9 (14.164 mg/100 g), while the RHE groups became unacceptable at day 12 (14.19 mg/100 g). The TVB-N level elevation driven by protein degradation and nitrogenous compound breakdown primarily comprised ammonia and primary/secondary/tertiary amines: compounds that promote microbial growth [45]. The decreased TVB-N level in rabbit meat might be explained by the TVC results (Figure 5C) in this study.

Microorganisms rapidly proliferate by utilizing the available nutrients, significantly elevating spoilage risk during refrigerated storage of pretreated rabbit meat. Further investigations revealed that HE treatment reduced TVC levels with prolonged storage time, as compared with those in the control groups. Yim et al. reported that *Caesalpinia sappan* L. extract showed the effectiveness of antimicrobial action in cooked pork sausages during storage [52]. Critically, hydroxyl groups of phenolics bind to microbial enzyme active sites via hydrogen bonds, inhibiting enzymatic function and inducing cell death [32]. Therefore, the inhibited microbial growth in HE-treated pre-treated rabbit meat might be due to the bioactive compounds of HE, which inhibited microorganisms and protected meat from spoilage. In addition, the decrease in TVC value was consistent with the TVB-N and TBARS results, indicating that HE treatment enhances antibacterial properties and extends rabbit meat freshness.

## 5. Conclusions

In summary, this study revealed the effects of HE supplementation on the quality of pre-treated rabbit meat during refrigerated storage. Firstly, honeysuckle extracted with 65% ethanol showed strong antioxidant activity and the optimal content of active compounds. In addition, HE treatment could delay the textural deterioration in pre-treated rabbit meat during refrigerated storage. Furthermore, HE treatment could inhibit the microbial growth and lipid oxidation in pre-treated rabbit meat during refrigerated storage. Thus, HE might have the potential to be used as an effective natural antioxidant and antimicrobial to improve quality loss and delay the refrigerated shelf-life of rabbit meat. In addition, further studies are required to explore the efficacy and dose of HE in rabbit meat.

## Figures and Tables

**Table 1:** Texture of raw and pre-treated rabbit with HE treatment during refrigerated storage.

	Storage Time (Day)	Hardness (g)	Elasticity (ratio)	Chewiness (g)	Cohesiveness (ratio)
RC	0	1625.84 ± 75.88 ^a^	0.72 ± 0.04 ^a^	949.51 ± 15.61 ^a^	0.65 ± 0.02 ^a^
	3	1346.31 ± 183.29 ^a^	0.65 ± 0.04 ^a^	791.14 ± 20.73 ^b^*	0.55 ± 0.04 ^a^
	6	1213.90 ± 133.92 ^a^	0.65 ± 0.04 ^a^	659.50 ± 18.61 ^c^*	0.51 ± 0.04 ^a^
	9	1183.48 ± 180.99 ^a^	0.65 ± 0.05 ^a^	479.96 ± 46.64 ^d^*	0.50 ± 0.05 ^a^
	12	1085.69 ± 207.64 ^a^	0.63 ± 0.04 ^a^	504.32 ± 16.63 ^d^	0.51 ± 0.05 ^a^
	15	1079.29 ± 101.34 ^a^	0.60 ± 0.03 ^a^	410.16 ± 33.49 ^d^	0.49 ± 0.02 ^a^
RHE	0	1762.13 ± 193.96 ^a^	0.69 ± 0.03 ^a^	932.34 ± 33.10 ^a^	0.65 ± 0.03 ^a^
	3	1717.08 ± 33.08 ^a^	0.69 ± 0.03 ^a^	877.88 ± 6.86 ^a^	0.58 ± 0.07 ^ab^
	6	1582.60 ± 37.92 ^a^	0.64 ± 0.04 ^a^	760.61 ± 35.40 ^b^	0.54 ± 0.02 ^ab^
	9	1571.21 ± 56.96 ^a^	0.62 ± 0.04 ^a^	632.41 ± 32.77 ^c^	0.46 ± 0.03 ^b^
	12	1459.47 ± 199.71 ^a^	0.61 ± 0.01 ^a^	579.93 ± 18.87 ^c^	0.43 ± 0.01 ^b^
	15	1233.67 ± 79.02 ^a^	0.60 ± 0.03 ^a^	441.76 ± 14.74 ^d^	0.44 ± 0.01 ^b^
CC	0	1939.41 ± 33.76 ^A^	0.66 ± 0.03 ^A^	969.36 ± 36.73 ^A^	0.62 ± 0.04 ^A^
	3	1776.18 ± 59.89 ^A^	0.67 ± 0.09 ^A^	827.40 ± 34.67 ^AB^*	0.55 ± 0.01 ^A^
	6	1630.84 ± 80.05 ^AB^	0.58 ± 0.02 ^A^	558.91 ± 19.10 ^BC^*	0.53 ± 0.02 ^A^
	9	1548.04 ± 128.52 ^ABC^	0.56 ± 0.02 ^A^	596.64 ± 50.33 ^BC^	0.53 ± 0.08 ^A^
	12	1290.29 ± 73.72 ^BC^	0.54 ± 0.01 ^A^	486.59 ± 30.62 ^C^	0.64 ± 0.01 ^A^
	15	1185.29 ± 44.55 ^C^	0.56 ± 0.01 ^A^	389.40 ± 7.25 ^C^	0.59 ± 0.04 ^A^
CHE	0	1936.80 ± 87.47 ^A^	0.61 ± 0.01 ^A^	1097.23 ± 30.74 ^A^	0.68 ± 0.01 ^A^
	3	1887.09 ± 116.62 ^A^	0.60 ± 0.02 ^A^	1097.59 ± 132.71 ^A^	0.53 ± 0.02 ^B^
	6	1789.55 ± 128.22 ^AB^	0.61 ± 0.04 ^A^	838.76 ± 176.49 ^AB^	0.57 ± 0.01 ^AB^
	9	1659.84 ± 105.22 ^AB^	0.59 ± 0.005 ^A^	546.80 ± 13.25 ^BC^	0.62 ± 0.01 ^AB^
	12	1390.89 ± 73.27 ^BC^	0.58 ± 0.05 ^A^	483.80 ± 57.14 ^C^	0.64 ± 0.08 ^AB^
	15	1155.29 ± 56.66 ^C^	0.59 ± 0.03 ^A^	408.37 ± 26.14 ^C^	0.62 ± 0.02 ^AB^

Note: Data are presented as means ± SE. (*N* = 3 per group). Different lowercase letters (a–d) in the same column indicate the significant differences within the different storage time (*p* < 0.05) among groups, RC vs. RHE. Different uppercase letters (A–C) in the same column indicate the significant differences within the different storage time (*p* < 0.05) among groups, CC vs. CHE. * Values in the same column within the same treatment indicate significant differences (*p* < 0.05). RC, control for raw; CC, control for cooked; RHE, HE for raw; and CHE, HE for cooked.

## Data Availability

The original contributions presented in the study are included in the article, further inquiries can be directed to the corresponding author.
